# Relevance of Endoplasmic Reticulum Stress Cell Signaling in Liver Cold Ischemia Reperfusion Injury

**DOI:** 10.3390/ijms17060807

**Published:** 2016-05-25

**Authors:** Emma Folch-Puy, Arnau Panisello, Joan Oliva, Alexandre Lopez, Carlos Castro Benítez, René Adam, Joan Roselló-Catafau

**Affiliations:** 1 Experimental Pathology Department, Instituto de Investigaciones Biomédicas de Barcelona, Spanish Research Council (IIBB-CSIC), Rosselló 161, 08036-Barcelona, Catalonia, Spain; efpbam@iibb.csic.es (E.F.-P.); arnau.panisello@iibb.csic.es (A.P.); 2 Department of Medicine, LaBioMed at Harbor UCLA Medical Center, Torrance, 90502 CA, USA; joliva@labiomed.org; 3 Centre Hépatobiliaire, AP-HP Hôpital Paul Brousse, Inserm U935, Université Paris-Sud, Villejuif, 75008 Paris, France; alexandregl.lopez@gmail.com (A.L.); ccastrob@gmail.com (C.C.B.); rene.adam@pbr.aphp.fr (R.A.)

**Keywords:** endoplasmic reticulum stress, unfolded protein response, liver ischemia reperfusion injury, orthotopic liver transplantation, University Wisconsin (UW) solution, Institut Georges Lopez-1 (IGL-1) solution

## Abstract

The endoplasmic reticulum (ER) is involved in calcium homeostasis, protein folding and lipid biosynthesis. Perturbations in its normal functions lead to a condition called endoplasmic reticulum stress (ERS). This can be triggered by many physiopathological conditions such as alcoholic steatohepatitis, insulin resistance or ischemia-reperfusion injury. The cell reacts to ERS by initiating a defensive process known as the unfolded protein response (UPR), which comprises cellular mechanisms for adaptation and the safeguarding of cell survival or, in cases of excessively severe stress, for the initiation of the cell death program. Recent experimental data suggest the involvement of ERS in ischemia/reperfusion injury (IRI) of the liver graft, which has been considered as one of major problems influencing outcome after liver transplantation. The purpose of this review is to summarize updated data on the molecular mechanisms of ERS/UPR and the consequences of this pathology, focusing specifically on solid organ preservation and liver transplantation models. We will also discuss the potential role of ERS, beyond the simple adaptive response and the regulation of cell death, in the modification of cell functional properties and phenotypic changes.

## 1. Introduction

The endoplasmic reticulum (ER) is an important intracellular organelle mainly responsible for the synthesis, folding and trafficking of a wide variety of proteins, including hormones, enzymes, receptors, ion channels and transporters. Under normal situations, a homeostatic equilibrium exists between the influx of unfolded peptides and the folding ability of the ER. However, when unfolded or misfolded proteins are accumulated in the organelle, endoplasmic reticulum stress (ERS) occurs and a specific adaptive response from mammalian cells is triggered to prevent this abnormal accumulation of unfolded proteins. ERS activates intracellular signal transduction pathways which together are known as the unfolded protein response (UPR) [1]. This response is characterized by three phases: an adaptive phase, an alarm phase and finally, a phase of cell death activation. The adaptive phase is characterized by the proteasome degradation of proteins and the subsequent overall inhibition of protein transduction that leads to increases in the chaperone pool and protein enzymes such as X box binding protein 1 (XBP-1) and activating transcription factor 4 (ATF4) [2]. This process limits ERS by reducing the load of misfolded proteins. The next step is the alarm phase, consisting of the induction of an inflammatory process by activation of Nuclear Factor-κB and c-Jun N-terminal kinases pathways. Ultimately, if these mechanisms are insufficient to relieve the load of misfolded protein, ERS activates an immune response against cell stress and eventually promotes at last the cell death [3].

Ischemia/reperfusion injury (IRI) of the liver graft is considered as one of crucial problems complicating post-transplant patient care and influences short- and long-term outcomes after liver transplantation. Since surgical techniques of transplantation obligate cold preservation and warm reperfusion of grafts, inevitable IRI occurs in every case of liver transplantation. The cellular damage in the hypoxic organ (ischemia-cold preservation) is worsened following the restoration of the oxygen supply after reperfusion [4]. Besides, it is known that hepatocytes are exceptionally active in synthesis of protein and lipids for export. As the result of these activities, ultrastructural examination of these cells reveals abundant quantities of both rough and smooth ER. Hence, one may consider that UPR/ERS response contribute either in preventing or mediating pathological changes in liver diseases. In this sense, there is increasing evidence that ER perturbations are novel subcellular effectors involved in the cold IRI associated with liver transplantation [5]. Certainly, ischemia reperfusion insult stimulates an overload of intracellular Ca^2+^ from the ER lumen which in turns modulates mitochondrial calcium and hepatocyte vulnerability to apoptosis, followed by cytochrome c release and caspase activation [6,7]. Once ER homeostasis is exacerbated, new unfolded proteins are accumulated and UPR is activated. This occurs during cold storage of the liver graft in preservation solutions; UPR/ERS alterations are then increased after reperfusion, which are determinant for the graft outcome after transplantation.

## 2. Key Players in the Endoplasmic Reticulum Stress (ERS) Response

In mammals, when the protein component of the ER increases, the signaling pathways of the UPR are mediated by three ER transmembrane proteins: activating transcription factor 6 (ATF6), inositol-requiring enzyme 1 (IRE1), and the PKR-like ER kinase (PERK). Under unstressful situations, the luminal domains of these sensor proteins are captured by ER intraluminal 78 kDa glucose-regulated protein (GRP78), also known as immunoglobulin-binding protein (BiP) [8]. Either by glucose deprivation, by the depletion of calcium stores or by the accumulation of misfolded proteins, the binding of GRP78 with misfolded and unfolded proteins is increased in ERS conditions, causing its dissociation from ATF6, IRE1 and PERK; this dissociation results in the release of these proximal ERS sensors and activates the UPR [9].

We next briefly describe the three main mediators involved in the signaling pathway of the UPR: IRE1, PERK, and ATF6.

### 2.1. Inositol-Requiring Enzyme 1 (IRE1)

IRE1 is the most highly conserved branch of the UPR among mammals. Its cytoplasmic tail has two enzymatic activities: a serine/threonine kinase domain and an endoribonuclease (RNase) domain [10]. Following the binding of misfolded proteins in the lumen, IRE1’s kinase is activated and *trans*-phosphorylates multiple serine/threonine residues on the cytosolic tail [11]. This phosphorylation causes the splicing of XBP1 mRNA. Spliced X box binding protein 1 (XBP1s) translocate to the nucleus to positively control the transcription of ER-resident chaperones, the genes involved in lipogenesis, the ER-associated protein degradation (ERAD) mechanism and ER quality control [12]. IRE1 signaling and XBP1 splicing are especially important in highly secretory cells where the machinery for protein folding is incessantly engaged with a high amount of nascent proteins [13]. Therefore, this branch of control serves as a key adaptive mechanism to match ER folding capacity with the demands of protein folding [14].

### 2.2. PKR-Like ER Kinase (PERK)

PERK is a type I ER transmembrane protein with serine/threonine kinase activity in its C-terminal cytosolic domain. Activation of PERK entails homo-dimerization and auto phosphorylation, leading to the phosphorylation of elongation initiation factor 2α (eIF2α). This phosphorylation inactivates eIF2α activity and consequently, decreases global protein translation [15]. This leads to the reduction of the ER protein-folding load. As an exception, ATF4 is selectively upregulated when the amount of active eIF2α is limited, and participates in the transcription of genes involved in amino acid metabolism, protein folding and autophagy [16].

### 2.3. Activating Transcription Factor 6 (ATF6)

ATF6 is a leucine zipper protein-containing transcription factor and a type II ER transmembrane protein. In the presence of misfolded proteins, ATF6 dissociates from GRP78, travels to the Golgi apparatus and is cleaved by the Site-1 and Site-2 proteases [17]. As a result, an N-terminal cytosolic domain of ATF6 (ATF6 (N)) translocate to the nucleus, induces the transcription of a number of UPR target genes (ER chaperones, transcription factors, components of ERAD and ER biogenesis) and increases its protein-folding capacity to promote cell survival [18].

Together, these proteins induce a cascade of cell signals that decrease the accumulation of misfolded proteins in the ER by upregulating ER chaperones expression, by inhibiting protein entry into the ER and by exacerbating the retrograde export of proteins from the ER to the cytosol for ubiquitination and degradation by the ubiquitin-proteasome system (UPS) [19]. Furthermore, because ERS can induce autophagy, this could activate other mechanisms in order to remove unfolded proteins independently of the UPS.

In the next part of this review, we describe the relevance of the UPR/ERS changes that occur during liver cold ischemia-reperfusion injury. We focus mainly on aspects that concern strategies of liver graft conservation using static preservation (organ preservation solutions) or machine perfusion (perfusate solutions), and we assess their relevance in liver transplantation when complete and reduced liver grafts are used.

## 3. Unfolded Protein Response (UPR)/ERS in Liver Graft Preservation: The Importance of Preventing ATP Breakdown

Cold storage protects organs by slowing their metabolism but also causes damage which is aggravated during cold conservation in the commercial preservation solutions used [20]. It is well established that hypothermia is a major cause of primary graft non-function, and new commercial preservation solutions are being developed in order to overcome this problem [21]. The introduction of University Wisconsin (UW) solution substantially improved graft preservation and consequently increased patient survival [22]. UW is the gold standard solution, but a limitation is that it contains hydroxy-ethyl starch (HES) as oncotic agent, conferring on it a high viscosity [23]. Institut Georges Lopez-1 (IGL-1) solution is a later development characterized by the substitution of HES by Polyethylenglycol-35 (PEG-35) and the reversal of the ionic K^+^/Na^+^ concentration [24]. In addition, other solutions with no oncotic agent have been formulated such as Celsior and histidine-tryptophan-ketoglutarate (HTK), which are used for liver transplantation [25,26]. Table 1 summarizes the differences between the organ preservation solutions [27,28,29].

During cold storage, the organ is deprived of oxygen and an energy breakdown occurs which is characterized by ATP depletion [30]. This provokes ischemic injury, which will be further aggravated during reperfusion. ATP depletion is responsible for the activation of 26S proteasome (a well-known ATP-dependent enzyme) and of the UPR. This confirms the importance of preventing energy breakdown during organ cold storage in which the intracellular and extracellular conditions interfere with ER function. This interference is associated with the accumulation of unfolded proteins in the ER, resulting in ERS, which will be further aggravated during reperfusion.

A recent study of the heart reported that a subset of the 26S proteasome is activated at critically low ATP concentrations. This activated subset was found to contribute to myocardial injury during cold ischemia, suggesting that proteasome inhibition during hypothermic organ preservation helps to prolong myocardial viability [31]. This hypothesis was confirmed by the use of proteasome inhibitors to prevent cardiac proteasome dysfunction during cold storage and reperfusion in a heart transplantation model in rats [32]. These investigations suggest that the use of proteasome inhibitors may help to maintain the physiological ubiquitin-protein conjugate pool during cold storage in organ preservation solutions, thus prolonging liver graft conservation and preventing subsequent cold IRI.

Besides this ATP depletion, during cold ischemia other cyto-protective factors such as adenosine mono phosphate protein kinase (AMPK) are activated as a self-response of the organ to oxygen deprivation. AMPK is stimulated in response to different stress factors in order to restore cellular and whole-body energy balance. This enzyme is regulated by competitive binding of AMP and ATP, through sensing cellular energy condition and, when activated, triggers a compensatory mechanism of ATP generation at the same time that attenuates ATP-consuming processes [33]. AMPK is degraded by the UPS and this degradation is blocked by the use of 26S proteasome inhibitors.

With this in mind, the use of preservation solution appears to be a useful tool for modulating some protective cell signaling pathways in cold preservation conditions that interfere with UPR/ER function and ERS alterations. This includes protective cell signaling pathways such as AMPK and nitric oxide generation, which are closely associated with the prevention of hepatic cold IRI. In this sense, we have recently reported that the inhibition of AMPK induced an increase in ERS and a significant attenuation in autophagy. These data confirm the close relationship between AMPK activation and ER stress and autophagy after cold IRI [34].

### 3.1. UPR/ERS in Liver Graft Cold Storage

Recently data from the European Liver Transplant Registry [35] suggested that UW, IGL-1 and Celsior solutions are the best alternative for use in liver transplantation and are superior to HTK. The presence of the oncotic agents HES (in UW) and PEG-35 (in IGL-1) characterize UW and IGL-1 solutions, while HTK and Celsior do not contain oncotic agents (see Table 1).

IGL-1 solution prevents ATP depletion more efficiently than UW after 24 h cold storage and consequently the activation of UPR is lower than when using UW [36]. Similarly, IGL-1 obtained a more significant AMPK activation than UW, attributed, in part, to the substitution of HES by PEG-35. Taking this into account and in view of the benefits of proteasome inhibition in hypothermic heart preservation [31], we recently explored the use of proteasome inhibitor as additives in UW and IGL-1 solutions for the purposes of liver preservation and transplantation [37]. As it is known that liver proteolysis during cold storage influences graft outcome after transplantation [38], the supplementation of UW and IGL-1 solutions with proteasome inhibitors such as MG132 and bortezomib (BZ) at low, non-toxic doses protected steatotic liver grafts against cold IRI. The inhibition of UPS activity was more effective with BZ (reversible drug action) than with MG132 (irreversible drug action) when livers were preserved in UW solution. These benefits were increased when BZ was used as an additive in IGL-1 solution, associating this solution as more effective in the prevention of proteolysis. In this case, the protective mechanisms were independent of those observed for UW. In the case of BZ, e-NOS activation promotes NO generation, which contributes to counterbalancing the exacerbated microcirculation alterations that occur in fatty livers after graft revascularization and transplantation. These findings corroborate the previous publications by Majetschak *et al.* in heart [39] confirming that the use of proteasome inhibitors may contribute to maintain the physiological ubiquitin-protein conjugate pool in liver grafts during cold storage, thus prolonging their preservation.

In another study, we analyzed the role of melatonin and trimetazidine combination as additives to IGL-1 solution in the modulation of ERS and autophagy in fatty liver grafts [34]. Both additives showed a protective effect by reducing ERS markers GRP78, phosphorylated PERK, and C/EBP homologous protein (CHOP) activation after reperfusion in accordance with an enhanced induction of autophagic parameters (beclin-1, ATG7, and LC3B) and AMPK phosphorylation.

### 3.2. UPR/ERS in Liver Graft Machine Perfusion

Simple cold storage and machine perfusion (MP) are the two current strategies to preservation before transplantation. Simple cold storage’s simplicity, low cost, and need for transport make it preferential at the majority of transplant centers. Nevertheless, MP includes a range of promising techniques of liver graft preservation that is currently making the transition into clinical practice.

Recently, it has been reported that the use of MP increases graft function and survival at one year compared to conventional hypothermic preservation [40]. However, when investigating the impact of MP with different preservation solutions on ERS of liver grafts from non-heart beating donor rats, Minor, *et al.* found that prolonged MP (more than 18 h) induced ERS-associated gene responses [41].

In this context, the occurrence of ERS responses after prolonged hypothermic MP might be lied to MP-dependence on the preservation solution used when HTK (a substrate free, low viscosity, extra-cellular type medium) was compared to MP Belzer-MPS (a nutritive, high viscosity, colloidal intra-cellular type solution), the most used in MP. It is important to remark that the differences could be associated to the presence of oncotic support on their compositions. HTK does not contain oncotic agent when compared to MP-Belzer.

With this in mind, the use of reversible UPS inhibitors as additives to Belzer-MPS gluconate solution or its generics at low, non-toxic concentrations could be useful for normothermic MP. In any case, a final short perfusion with Belzer-MPS alone may be envisaged in order to eliminate BZ molecule (reversible UPS inhibitor) traces. The fact that BZ promotes NO generation and AMPK activation, and also contributes to up regulates liver antioxidative enzymes [42] suggests that this practice might also help to reduce the fat in steatotic livers (those with more than 60% steatosis) and recuperate them for the organ pool.

## 4. UPR/ERS in Liver Transplantation

Liver transplantation has been considered as the best therapeutic treatment for the patients with end stage liver disease, including advanced liver cirrhosis and acute liver failure. In any case, IRI is inevitable and contributes to the early liver graft non-function or late dysfunction, limiting successful outcome after transplantation.

Distinct ERS responses are triggered during human liver transplantation. Samples from ischemic and reperfused livers, showed a biphasic activation of UPR pathways [42]. The early ischemic phase initiated the activation of IRE1α which was further increased upon reperfusion. In addition, ischemic hepatocytes showed lessened PERK and eIF2α phosphorylation being then enhanced during reperfusion mainly in sinusoidal endothelial cells. On the other hand, ERS has been recently proposed as a marker for predicting steatotic liver outcome after transplantation [43]. The study suggests that the ERS pathways, particularly the CHOP-caspase 11-Interleukin1β pathway, are potential targets to improve steatotic liver allograft function following liver transplantation.

The consequences of UPS/ERS in liver transplantation have been poorly investigated; especially with regard to the use of different preservation solutions (see Table 1). The compositions of the solutions used for graft conservation are crucial for preventing ATP breakdown and for promoting a differential activation of UPR/ERS after liver transplantation. We have reported that IGL-1 solution prevented ERS more efficiently than UW by reducing the activation of three pathways of the UPR (IRE1, PERK and ATF6), as well as their effector molecules caspase 12-CHOP, XBP-1, tumor necrosis factors-associate factor 2 and eukaryotic translation initiation factor 2 [37]. This was associated with a reduction in liver injury and apoptosis.

The mechanisms by which IGL-1 confers better protection against ERS and maintains cell viability are directly related to the prevention of ischemic (activation of UPR by ATP during cold storage) and reperfusion injury (prevention of oxidative stress and ERS), injuries which are exacerbated during graft revascularization. The presence of PEG-35 in IGL-1 solution appears to be a key to promoting cyto-protective factors like AMPK and NO. PEG-35 may also potentiate the benefits of UPS inhibitors and provide an extra protection for the liver graft against cold IRI.

Because of the lack of organs available for transplantation, living donor liver transplantation (LDLT) has emerged as an alternative to increase the number of transplantable organs. Although IRI is also inevitable, the ischemia injury is clearly reduced when compared to cadaveric donors but the grafts may also be non-functional due to the appearance of small-for-size liver syndrome (SFSS) which compromises the liver outcome after transplantation [44]. In this case, the prevention of IRI is even more important in order to obtain rapid liver regeneration, to achieve a suitable critical liver mass after transplantation and to avert SFSS.

## 5. Proteasome Inhibitors as Therapeutic Targets for Liver Preservation and Transplantation

The proteasome plays a significant role in not only cell protein degradation, but also in the proteolysis of misfolded proteins in the ER. In this sense, reversible UPS inhibitors appear as therapeutic targets for liver preservation in static and dynamic conditions and as potential drugs in liver transplantation. The development of new reversible UPS inhibitors may increase the efficacy of dynamic preservation in cold or normo-thermic conditions. They may also be used as pretreatment for liver transplantation, as potential therapeutic especially at a non-toxic single low dose.

As mentioned before, the supplementation of UW and IGL-1 preservation solutions with proteasome inhibitors such as MG132 and BZ protects steatotic liver grafts against cold IRI being BZ more effective than MG132 [37]. The protective effects of this reversible inhibitor have been found to be partially mediated through the activation of AMPK and Akt/mTOR signaling.

Regarding the effect of proteasome inhibitors upon experimental liver transplantation, the pretreatment of rat recipients and donors before reduced-size orthotopic liver transplantation (ROLT) with BZ and MG123 at single low non-toxic dose have been found to protect small liver grafts [45]. BZ protection is superior to MG132 treatment in ROLT, inducing an overwhelming protection of mitochondrial damage concomitantly with effective prevention of oxidative stress, ERS and apoptosis, especially when IGL-1 was used instead of UW [37]. In terms of hepatic regeneration, BZ also performed better than MG132 and promoted a better hepatocyte proliferation [45].

Another mechanism of action of BZ has been found in transplanted steatotic grafts. Pretreatment with BZ resulted in significant decrease in NF-κB activation, leading to the reduction of the levels of pro-inflammatory cytokines [46].

**Figure 1:**
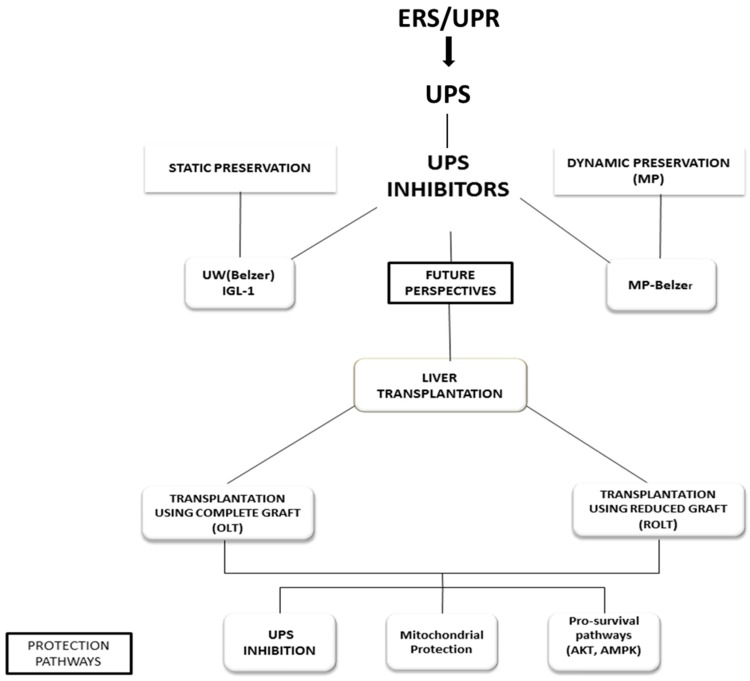
New perspectives to use ubiquitin-proteasome system (UPS) inhibitors as additives in static (University Wisconsin (UW) and Institut Georges Lopez-1 (IGL-1) solutions) and dynamic machine perfusion (MP) (MP Belzer solution) liver preservation; as well as in liver transplantation using complete (OLT) and reduced grafts (ROLT). In all cases, the protection mechanisms are associated with the UPS inhibition, mitochondrial protection and activation of pro-survival factors (Akt, adenosine mono phosphate protein kinase (AMPK)). ROLT, reduced-size orthotopic liver transplantation.

Apart from BZ and MG132, other proteasome inhibitors such as lactacystin and epoxomicin, have been used to limit IRI in different organs [31,47,48,49]. Both of these compounds inhibit proteasome activity, and, additionally epoxomicin prevents the activity of the immunoproteasome, the key complex in the inflammatory response [50,51]. However, these compounds are not highly specific to proteasome and are irreversible inhibitors that exert more damage than protecting effects than those obtained with BZ treatment. The dose of BZ used in our study was within the ranges of the approved therapeutic concentration in clinical trials for the treatment of multiple myeloma and mantle cell lymphoma. The potential benefits of UPS inhibitors for limiting liver cold IRI are summarized in Figure 1.

## 6. Other Therapeutic Targets for Liver Preservation and Transplantation

The maximum concentrations of calcium within the cell are found in the ER due to the active transport by Ca^2+^-ATPases in this organelle. Therefore, derangements of calcium regulation in the ER also contribute to the problems related with protein unfolding because of the calcium-dependent nature of some chaperones such as GRP78 and calreticulin 3 [52]. In case of severe and sustained ERS, calcium release from the ER via IP3 receptors can activate calpains, a family of Ca^2+^-dependent cysteine proteases, implicated in the subsequent cell death through different substrates including Bax and Bid, Bcl-2 and Bcl-xL and several caspases. In this case, the blockade of calpain activation could be an interesting approach to decrease, at least, cell apoptosis after grafting organs such as liver. In experimental models of cardiac ischemia/reperfusion, the inhibition of calpain both at pharmacologic and genetic level have been already demonstrated to ameliorate the ischemic cardiac injury and improve myocardial function [53,54,55]. This cell death calcium-dependent can be amplified by the decrease of cellular ATP. Indeed, low ATP levels during ERS impair the storage of Ca^2+^ in ER and it is transported out of the cells [56].

Another source of Ca^2+^ release from the ER with the potency to ameliorate ERS is via ryanodine receptor, a calcium-release channel that controls the delivery of Ca^2+^ into the cytoplasm [57]. In this sense, the use of the ryanodine receptor antagonist Dantrolene in mice protected the ischemic liver through the modulation of inflammatory related cytokines Tumor Necrosis Factor alpha and Interleukin 10 [58].

## 7. Conclusions

In summary, the involvement of UPR/ERS in solid organ transplantation is probably underestimated. Modulation of these processes is crucial for preventing the cold IRI associated with liver transplantation when using either complete or reduced grafts. The use and development of proteasome inhibitors for future therapeutic applications in clinical transplantation need to be explored further, placing special emphasis on their potential use as additives in static preservation and MP solutions. UPS inhibitors and other compounds with potency to ameliorate ERS may also be a useful tool for recovering some of the fatty livers for transplantation, which are currently discarded, thus increasing the number of transplantable organs for patients on waiting lists.

## Figures and Tables

**Table 1:** Chemical composition of liver graft preservation solutions.

Solution Components	University of Wisconsin Solution (UW)	Institute George Lopez 1 (IGL-1)	HTK Custodiol
Electrolytes in mM			
K^+^	125	25	9
Na^+^	30	125	15
Mg^2+^	5	5	4
Ca^2+^	0	0	0.015
Cl^−^	0	0	32
SO_4_^2−^	5	5	0
Buffers in mM			
Diphosphate	25	25	0
Histidine	0	0	180
Histidine-HCl	0	0	18
Tryptophan	0	0	2
Non-Permeants in mM			
Raffinose	30	30	0
Lactobionic Acid	100	100	0
Mannitol	0	0	30
Colloids in g/L			
Hydroxyethyl Starch	50	0	0
Polyethylene Glycol-35	0	1	0
Antioxidants in mM			
Glutathione	3	3	0
Allopurinol	1	1	0
Metabolic Precusors in mM			
Adenosine	5	5	0
Ketoglutarate	0	0	1
pH	7.4	7.4	7.2
Osmolarity in mOsmol/L	320	290	310

## Abbreviations

The following abbreviations are used in this manuscript:

ER	endoplasmic reticulum
ERS	endoplasmic reticulum stress
UPR	unfolded protein response
IRI	ischemia-reperfusion injury
UPS	ubiquitin proteasome system
ATF6	activated transcription factor 6
IRE1	Requiring enzyme inositol 1
PERK	RNA protein kinase (PKR) -like ER kinase
GRP78	78 kDa glucose-regulated protein
BiP	immunoglobulin-binding protein
AMPK	adenosine mono phosphate protein kinase
HIF1 alpha	hypoxia inducible factor 1 alpha
XBP-1	X box binding protein 1
ERAD	Endoplasmic reticulum associated protein degradation
eIF2a	elongation initiation factor 2a
ATF4	activating transcription factor 4
UW	University of Wisconsin solution
IGL-1	Institut Georges Lopez-1
PEG-35	Polyethylenglycol-35
HTK	histidine-tryptophan-ketoglutarate
BZ	Bortezomib
CHOP	C/EBP homologous protein
Belzer-MPS	Belzer gluconate solution
MP	Machine perfusion
LDLT	Living donor liver transplantation
ROLT	reduced-size orthotopic liver transplantation
SFSS	Small-for-size liver syndrome
